# Supplementary material for: Cost-effectiveness of Hepatitis C virus self-testing in four settings
Source: PLOS Glob Public Health. 2023 Apr 5;3(4):e0001667. doi: 10.1371/journal.pgph.0001667 (PMC10075433; doi:10.1371/journal.pgph.0001667)
Supplement: Attachment — Submitted filename: Response to reviewers.docx [file pgph.0001667.s003.docx]

# Response to reviewers

Thank you for taking the time to review this paper. We have addressed all of the comments from the editors and reviewers as outlined below in red text. We believe the paper has been significantly improved due to the changes described below.

## Editorial comments:

1. Please amend your detailed Financial Disclosure statement. This is published with the article. It must therefore be completed in full sentences and contain the exact wording you wish to be published. a. State the initials, alongside each funding source, of each author to receive each grant.b. State what role the funders took in the study. If the funders had no role in your study, please state: “The funders had no role in study design, data collection and analysis, decision to publish, or preparation of the manuscript.”

If you did not receive any funding for this study, please simply state: “The authors received no specific funding for this work.”

***Response:*** We have revised the funding statement as follows: Unitaid and the Netherlands Government funded this study as part of the HEAD-Start project led by SS at FIND. PV also acknowledges support from the NIHR Health Protection Research Unit in Behavioural Science and Evaluation at the University of Bristol. The funders had no role in study design, data collection and analysis, decision to publish, or preparation of the manuscript.

2. Please provide separate figure files in .tif or .eps format only and remove any figures embedded in your manuscript file. Please also ensure that all files are under our size limit of 10MB.

For more information about figure files please see our guidelines:  LINK

<https://journals.plos.org/globalpublichealth/s/figures> <https://journals.plos.org/globalpublichealth/s/figures#loc-file-requirements>

***Response:*** We have uploaded files as TIF at 300 dpi after converting figures through PACE.

3. We do not publish any copyright or trademark symbols that usually accompany proprietary names, eg ©, ®, or ™  (e.g. next to drug or reagent names). Please remove all instances of trademark/copyright symbols throughout the text, including ® on page 9.

***Response:***  We have removed these symbols.

4. We have noticed that you have uploaded Supporting Information files, but you have not included a list of legends. Please add a full list of legends for your Supporting Information files after the references list.

***Response:*** We have added a list of legends at the end of the manuscript file.

## Reviewer comments:

### Reviewer #1:

The authors incorporated most of the changes requested in the previous review, and the article meets all the technical requirements to be published. However, the originality of the work in the field of knowledge is low.

***Response:*** I sent a follow up to the editors to clarify this comment, as it does not apply to this paper which has not previously been reviewed. The editors said they would follow up with the reviewer, but I have not yet received any further information. Therefore we have not made any changes based on this comment.

### Reviewer #2:

This paper looks at the cost and cost-effectiveness of an emerging technology to increase diagnosis and treatment uptake for hepatitis C. The study is timely given the need to engage new populations in hepatitis C care, and will be useful to guide decisions on how to test people not engaged in care. The methods are suitable for the study aims, appropriate sensitivity analyses are conducted and the manuscript is clear and well written. While there are some assumptions, they are reasonably justified based on HIV and other experiences and necessary in lieu of pilots. I have some minor points below.

***Response:*** Thank you for taking the time to thoughtfully review this paper.

1. There is not a lot of information provided on the cost estimates. E.g. it is noted that they are for HCV testing and treatment, and include overheads, staff time, training, outreach, facilities, and start-up costs. Some further details could be provided on the breakdown of costs, rather than single totals.

***Response:*** All costs were gathered from previous studies, and we have clarified the methods as follows: “Costs were identified from previous studies for HCV testing and treatment, and/or HIV self-testing in each setting from the healthcare providers’ perspective. We aimed to identify costs which accounted for overheads, staff time, training, outreach, facilities, and start-up costs where available, however, costing methodology may vary by source.” In addition, we have updated several references for cost data which were previously in press to their final published versions.

2. The outcome measures are based on cost per diagnosis and cost per treatment, which is not a problem but can be difficult to benchmark. Perhaps in Table 3 it would be useful to report the baseline cost per diagnosis and cost per treatment (i.e. without self-testing)? This information can be obtained from the table, so it is just being more explicit.

***Response:*** Thank you for this suggestion. We agree this is important information to include, and it is in the text in the second section of the results. In order to make this clearer, we have switched the order of the main text so that the baseline in the absence of HCVST is presented first, followed by the result for introducing HCVST.

3. In Table 3, are the numbers diagnosed and treated in each year influenced by the stage of elimination that these countries are up to? In the text it is noted that these are influenced by population size and prevalence, but also depending on how long DAAs have been available these numbers may be abnormally high or may have stabilized.

***Response:*** This is a good question. The availability of treatment in each setting certainly has an influence on these numbers, which are affected by the whole cascade of care from the proportion undiagnosed, standard of care test uptake, treatment uptake, cure rate, etc. We have added a sentence to the limitations section of the discussion as follows: “The current availability of testing and treatment for HCV in each setting will also affect the observed cascades of care identified, and these rates are likely to change over time as each setting progresses towards elimination.”

### Reviewer #3:

Reviewer comments to manuscript "Cost and cost-effectiveness of Hepatitis C virus self-testing in four settings: an economic evaluation" The title is repetitive it should not include the words “… an Economic evaluation” and “Cost” since a cost effectiveness is an economic evaluation study. In addition, it is implicit that a cost effectiveness study includes an estimate and analysis of costs.

***Response:*** We have revised the title to be “Cost-effectiveness of Hepatitis C virus self-testing in four settings”.

On Figure 1: The decision tree requires to be reported as in conventional notation showing decision and probabilistic nodes. In addition, highlight the probabilistic values assumed in estimating the expected value of number of diagnosed and cure cases as well as the costs.

***Response:*** We have updated Figure 1 to use conventional notation for decision trees and updated the figure legend, we believe this is now much easier to interpret. Due to the size of the tree and that probabilistic values and costs vary for each of the four settings, we have not included these values within the decision tree diagram. They are presented in Table 1 and Supplementary Tables 1 and 2, which we have also clarified in the figure caption.

In the alternative of no tested in the decision tree it is not clear whether this is a relevant alternative to be evaluated since the relevant comparator of the value of HCVST is not to use it. In addition, if the relevant outcome is to be aware of the HCV status that means that being positive or negative is not the relevant outcome but to be aware of its health status. Seems to be confusing as it is reported in the current manuscript, see decision tree from reference of Schackman BR 2015Schackman BR, Leff JA, Barter DM, DiLorenzo MA, Feaster DJ, Metsch LR, Freedberg KA, Linas BP. Cost-effectiveness of rapid hepatitis C virus (HCV) testing and simultaneous rapid HCV and HIV testing in substance abuse treatment programs. Addiction. 2015 Jan;110(1):129-43. doi: 10.1111/add.12754. PMID: 25291977; PMCID: PMC4270906.

***Response:*** Thank you for directing us to this reference. In this case, the comparator/counterfactual to introducing HCVST is to have only standard of care testing without HCVST. This is defined in line 172 of the methods “For each setting, we compared the introduction of HCVST … to a counterfactual standard of care scenario in which no self-testing occurs”. The study aim is to evaluate the incremental cost per diagnosis (confirmed active infection) and cost per cure. The potential for false positives or false negatives is accounted for according to the sensitivity and specificity of the tests. The rate of diagnosis therefore could be considered being aware of chronic infection, while false negatives would remain undiagnosed. We have clarified this in the updated decision tree diagrams (see response to previous comment) by adding the word diagnosed to the viraemic infection outcome.

Tables 3 and 4 should have some explaining nots to interpret results reported.

***Response:*** In order to clarify how the tables are structured, we have added to each of the legends “The incremental cost per [diagnosis/cure] for the HCVST scenario compared to no HCVST is presented for each of the four settings.” Interpretation of the results is in the text of the results, which we have also revised for clarity (see response to reviewer 2 point 2 above).

Table 3 specify these are US dollars 2019

***Response:*** We have revised the table legend for tables 3 and 4 to clarify that results are in 2019 US dollars.

Table 4 is supposed to include treatment costs modify title as corresponds

***Response:*** We have revised the table legend to clarify that these calculations include the cost of treatment.
