# Supplementary material for: Cost-effectiveness of Hepatitis C virus self-testing in four settings
Source: PLOS Glob Public Health. 2023 Apr 5;3(4):e0001667. doi: 10.1371/journal.pgph.0001667 (PMC10075433; doi:10.1371/journal.pgph.0001667)
Supplement: Attachment — Submitted filename: Response to reviewers R2.docx [file pgph.0001667.s004.docx]

# Response to reviewers

Thank you for taking the time to review this paper again. We have addressed all of the comments from the editors and reviewers as outlined below in red text. We believe the paper has been significantly improved due to the changes described below.

## Editorial comments

Please review your reference list to ensure that it is complete and correct. If you have cited papers that have been retracted, please include the rationale for doing so in the manuscript text, or remove these references and replace them with relevant current references. Any changes to the reference list should be mentioned in the rebuttal letter that accompanies your revised manuscript. If you need to cite a retracted article, indicate the article’s retracted status in the References list and also include a citation and full reference for the retraction notice.

**Response:** We have reviewed all of our references and updated as follows. None of the references have been retracted.

- Reference 10, Stone et al, was in press and now published in AIDS so we have updated the reference with full details.
- Reference 32 is a new reference for an additional recently published protocol of an HCV ST trial which is underway.

## Reviewer comments

### Reviewer #1:

Several problems with the model are observed. First, the authors assume that the four scenarios have the possibility of increasing care in medical units to confirm cases with the development of tests such as NAT. In addition, they consider that it is possible to provide medical care and treatment to the population diagnosed with self-diagnostic tests (human resources, infrastructure, and budget). Adopting more realistic or conservative coverage levels for each locality where the cost-effectiveness model is being carried out is suggested.

**Response:** Thank you for this suggestion. We agree that a key challenge of expanding screening and diagnostics for HCV will be making sure there is capacity to link additional diagnosed patients to care. Regardless of diagnosis route, if targets for treating HCV are to be met, treatment capacity will have to be scaled up. In our example settings, the biggest numerical increase in patients diagnosed is in Georgia, where 1,093 additional patients are diagnosed. Georgia has an ongoing national elimination program which aims to treat 150,000 people and has so far treated 70,000, so the capacity does exist there. We assumed that the cost of testing and treatment scales linearly with the number of diagnoses and treatments given within our one year time horizon (approximately 30% increase). We have added a sentence to the limitations section of the discussion in line 399-401 to emphasize this: “Our analysis assumes that there is capacity within the system for scaling up diagnosis and treatment, which ranges from an additional 3 diagnosed patients in the China setting to 1,093 additional patients in the Georgia setting in the base case, and that costs will scale linearly with the number of patients treated.”

Among the most robust assumptions that would have to be assumed is that the chain/sequence of care can be fulfilled by the health services that are being proposed.
Another situation to consider is the percentage of acceptance of the test and if people intend to take it to find out their condition.

**Response:** Thank you, we agree evaluating these assumptions is important. In sensitivity analysis, we varied parameters for proportion linking to care, uptake of HCV self-testing, and proportion of those using self-testing who would otherwise access facility-based tests. These are outlined in Table 2 and results presented in Figure 3 and page 15-16.

It is necessary to validate several of the parameters of the China scenario because it is the location with the highest cost. Also, find out if these costs are due to low availability or other reasons.

**Response:** The China scenario does not have the highest unit costs for testing, diagnosis, or treatment. The reason it has the highest cost per diagnosis is because of the low prevalence in the sample population. We emphasize the importance of prevalence in line 312 in the results, and line 365 in the discussion.

The modifications in the efficacy of the self-application tests are minimal, which can determine the non-variability or impact on the ICER. It is suggested to incorporate variations of effectiveness in actual care conditions for this type of test.

**Response:** The efficacy of the test was varied according to published efficacy rates observed in previous studies. We also varied linkage to care rates, uptake rates, substitution of facility-based tests, and proportion of invalid self tests.

Indeed, the authors did not calculate the future impact, but they did not calculate the probability of getting sick again. Additionally, due to the type of disease and population, it would be necessary to reapply for these tests annually.

**Response:** That is correct, we conducted this analysis with the aim of estimating the first year of implementing the HCV self tests. In a longer term model it would be necessary to incorporate re-testing for those who previously tested negative for HCV antibodies and are at high risk of infection. However, that consideration is beyond the scope of this study, which we outline in the discussion section lines 385-389.

Supplement 1
It is recommended to search for values published in the literature of other programs rather than applying their assumptions to these parameters.

**Response:** We are not certain what the reviewer is referring to in this comment, but think it relates to Supplementary Table 3 which has “Assumption” listed for several of the parameter sources. We have updated where possible to clarify sources. For the prices of the self tests, these were estimated based on expert opinion of some of the authors who have extensive knowledge of HCV diagnostics. The percent of facility-based tests replaced by self-tests is based on a model of HIV self testing, we have now added this reference which was missing. The percentage of invalid tests, we assume is 3% and vary in sensitivity analysis, is in addition to accounting for people reading the tests incorrectly, which we account for by the inter-reader agreement parameter, so we think this is a conservative estimate. The other assumptions are structural model assumptions: first, we assume 5% of those with a negative self test will come to a facility, but that none of them will receive a follow up test. We assume all patients who come with a negative or invalid test result will be offered a test. These have been left with the source labelled as “Assumptions” in Supplementary Table 3.

### Reviewer #2:

I have no further comments.

**Response:** Thank you for taking the time to review this paper again.

Reviewer #3:

2nd review round of the manuscript
Cost-effectiveness of Hepatitis C virus self-testing in four settings

**Response:** Thank you for thoroughly reviewing the manuscript and catching the errors you identify below. We have addressed them as described below.

General comments
1. It seems that figure 1 explanation in lines 143-153 have to be separated from title of the figure 1.

**Response:** We have updated to indicate that part of the caption should be a footnote to the figure.

2. In page 11 (lines 159) authors refer to figure 1A but if they replace the previous figure 1A and 1B with the new Figure 1 of the decision tree which is only one. Make corrections as correspond.

**Response:** We have corrected this to refer to Figure 1 and checked the rest of the paper to make sure there are no other lingering references to 1A or 1B.

Methodology
1. The probabilities of the decision tree are described in table 1 and supplementary tables 1-3. In the case of supplementary tables 1 and 2 the values are not reported because they vary by country?

**Response:** In order to make the table sizes more management, we split the parameters into multiple tables. Table 1 shows the parameters which vary by setting, for each setting. Supplementary Tables 1 and 2 show how the transition probability parameters were calculated and where costs are applied. Supplementary Table 3 includes all parameters which do not vary by setting.

Results and SA
1. Authors say in base case that with introduction of HCVST the number of individuals diagnosed will increase by 62%, is that a target or is it estimated from table 1?

**Response:** In the methods section line 202-205, we describe that the uptake and linkage to care parameters are based on randomised controlled trial data from HIV self testing, as no data is available on HCV self testing implementation yet. This assumes that there will be a 62% increase in the number of people tested for antibodies after the introduction of self testing. In the results section, as the reviewer identifies, we repeat that we’ve assumed introducing HCVST will increase testing by 62% in all settings, in order to contextualise that this leads to different numbers diagnosed or cured in each setting depending on their respective cascades of care.

2. There is a last comment on the sensitivity analysis. The lack of a probabilistic sensitivity when all parameters are varied for an estimate of the joint effect on cost effectiveness should be reported as a limitation of the study. Otherwise this analysis should be conducted.

**Response:** We agree this is a limitation of the study. The aim of the study was to explore scenarios for potential implementation of HCVST in different populations. As a result, we focused on one-way scenario analysis and accounting for structural uncertainty (such as whether patients would go direct to NAT or be re-tested for antibodies at the healthcare facility). We’ve added a sentence to the limitation section of the discussion in lines 378-381: “As this study focused on the potential implementation of HCVST in different settings under different structural assumptions, we focused on one-way sensitivity analysis, and did not include a probabilistic analysis of the joint effect of parameters on the cost-effectiveness.”

Discussion (Interpretation)
1. Authors say that in base case scenario the arm for self-testing includes 4 alternatives. For base case estimates this is not a problem: the model begins with self-test then positives are confirmed with serological test then positives with NAT. But this is not clear for sensitivity analysis. In SA the starting test is self-test then those positive go direct to NAT?

**Response:** In a previous draft of the paper we presented all outcomes for both repeat serological testing scenario and direct to NAT scenario, but here it has been simplified so that direct to NAT is just one of the scenarios explored in sensitivity analysis. We have clarified this in the text: In line 123 in the methods, repeat serological testing is the assumption of the model, and that direct to NAT is explored in sensitivity analysis. In line 163, we removed the description of the branch going direct to NAT from the model description, as this is not shown directly in Figure 1. In line 226, we added that each sensitivity analysis compares the introduction of HCVST to counterfactual of no self-testing.

The branch for unclear/unknown which go direct also to NAT? And then the idea is that additional cases are diagnosed from this group of unclear/unknown, something which the alternative of facility based test does not consider, in addition to other reasons?

**Response:** The unclear/unknown result does not ever go direct to NAT, we assume these patients come to the clinic having taken the self test but without being able to show what the result was. Therefore they would receive a facility-based test in all circumstances. We have clarified this in Table 2 first row under the heading (line 234).
